# GLP‐1 Receptor Agonists for the Prevention of New‐Onset Heart Failure: A Systematic Review and Meta‐Analysis of Placebo‐Controlled Randomized Clinical Trials

**DOI:** 10.1111/obr.70043

**Published:** 2025-11-25

**Authors:** João Sérgio Neves, Carolina B. Lobato, Ana Rita Leite, Catarina Vale, Francisco Vasques‐Nóvoa, Francisca Saraiva, Adelino Leite‐Moreira, Jens J. Holst, João Pedro Ferreira

**Affiliations:** ^1^ RISE‐Health, Department of Surgery and Physiology Faculty of Medicine of the University of Porto Porto Portugal; ^2^ Department of Endocrinology, Diabetes and Metabolism ULS São João Porto Portugal; ^3^ Section of Endocrinology, Department of Medicine Copenhagen University Hospital, Amager and Hvidovre, Section of Endocrinology Hvidovre Denmark; ^4^ Department of Biomedical Sciences, Faculty of Health and Medical Sciences University of Copenhagen Copenhagen Denmark; ^5^ Department of Internal Medicine ULS São João Porto Portugal; ^6^ Department of Cardiothoracic Surgery ULS São João Porto Portugal; ^7^ Inserm, Centre d'Investigations Cliniques, ‐ Plurithématique 14‐33, and Inserm U1116, CHRU Nancy, F‐CRIN INI‐CRCT (Cardiovascular and Renal Clinical Trialists) Université de Lorraine Nancy France

**Keywords:** glucagon‐like peptide‐1 receptor agonists, heart failure, obesity, type 2 diabetes

## Abstract

**Introduction:**

Glucagon‐like peptide‐1 receptor agonists (GLP‐1 RA) improve outcomes in heart failure (HF) with preserved ejection fraction. Whether GLP‐1 RA prevent new‐onset HF in Type 2 diabetes or obesity requires further investigation.

**Methods:**

We performed an updated meta‐analysis of randomized placebo‐controlled trials (RCT) of treatment with GLP‐1 RA in participants without HF. The hazard ratio (HR) and 95% confidence intervals (95% CI) were extracted from the group without HF in each study. The primary outcome was time to first HF event (HF hospitalization or urgent visit for HF). The correlation between the effect of GLP‐1 RA on HF events and the effects on HbA1c, weight and major atherosclerotic cardiovascular events (MACE) was also investigated. We also evaluated the heterogeneity of effect according to study characteristics.

**Results:**

A total of 52,752 participants without HF from six RCTs were included. Treatment with GLP‐1 RA (vs. placebo) decreased the risk of new‐onset HF (HR = 0.77 [95% CI 0.65–0.93], *p* < 0.001) and the composite of HF events or cardiovascular death (HR = 0.82 [95% CI 0.76–0.89], *p* < 0.001). The effect of GLP‐1 RA on HF events was independent of its effects on HbA1c or weight, but was correlated with its protective effects on MACE. The effects on HF prevention were more pronounced in studies restricted to patients with atherosclerotic cardiovascular disease and in trials with higher incidence rate of HF events.

**Conclusion:**

Treatment with GLP‐1 RA decreases the risk of new‐onset HF in patients with Type 2 diabetes or obesity.

## 1 Introduction

Type 2 diabetes and obesity are important risk factors for developing heart failure (HF) [1, 2, 3]. HF is a major cause of hospitalization, reduced quality of life, and early mortality [4]. Up to 22% of patients with Type 2 diabetes have HF [5], and patients with obesity have more than a 50% increased risk of developing HF, independently of other cardiovascular risk factors [6]. The concurrent presence of chronic kidney disease (CKD) further amplifies this risk, with individuals having CKD facing a two‐ to three‐fold greater likelihood of developing HF compared with those without CKD [7, 8]. Given its significant burden, preventing the onset of HF in these populations is of utmost importance. Some interventions including intensive blood pressure control, the use of SGLT2 inhibitors and the use of finerenone have shown a protective effect on the development of HF [1]. However, even after these interventions, the residual risk of HF remains high, underscoring the need for additional preventive strategies.

GLP‐1 receptor agonists (GLP‐1 RAs) improve glycemic control, reduce body weight, and reduce the risk of atherosclerotic cardiovascular events (ASCVD) [9, 10, 11]. Recent data from three large outcome randomized clinical trials (RCTs; Research Study to Investigate How Well Semaglutide Works in People Living With Heart Failure and Obesity [STEP‐HFpEF], Research Study to Look at How Well Semaglutide Works in People Living With Heart Failure, Obesity and Type 2 Diabetes [STEP‐HFpEF DM] and A Study of Tirzepatide in Participants With Heart Failure With Preserved Ejection Fraction and Obesity [SUMMIT]), specifically designed to evaluate the effects of GLP‐1 RA in HF and preserved ejection fraction (HFpEF), have shown a reduction in the risk of worsening HF events with GLP‐1 RA [12, 13]. A meta‐analysis of participants with HFpEF from the STEP‐HFpEF, the STEP‐HFpEF DM, the FLOW (A Research Study to See How Semaglutide Works Compared to Placebo in People With Type 2 Diabetes and Chronic Kidney Disease), and the SELECT (Semaglutide Effects on Heart Disease and Stroke in Patients With Overweight or Obesity) trials showed a 41% relative reduction in the risk of worsening HF events with semaglutide [14]. However, whether GLP‐1 RAs prevent HF events in patients without a previous history of HF requires further investigation.

Our group previously conducted a meta‐analysis examining the effects of GLP‐1 RA in patients with Type 2 diabetes with and without a history of HF [15]. In this analysis, we found that while the effects of GLP‐1 RA on atherosclerotic events were not influenced by HF history, the effect on HF hospitalization was modified by HF status, with a greater reduction in the risk of HF hospitalization among those without HF. However, this meta‐analysis was limited to patients with Type 2 diabetes, and all the included studies had a low prevalence of CKD. The recent publication of the SELECT and FLOW trials, which involved a large number of patients with obesity and CKD at high risk of developing HF, presents a unique opportunity to evaluate the effects of GLP‐1 RA on the prevention of new‐onset HF.

Our objective was to conduct an updated meta‐analysis and a meta‐regression analysis of the effects of GLP‐1 RA on HF outcomes in patients with Type 2 diabetes or obesity without known HF.

## 2 Material and Methods

### 2.1 Search Strategy and Selection Criteria

This meta‐analysis was conducted in accordance with the Preferred Reporting Items for Systematic Reviews and Meta‐analyses (PRISMA) reporting guideline [16]. We searched for published RCTs testing GLP‐1 RA and reporting data on participants without HF at baseline. We defined both MeSH terms (controlled language) and free text terms to express each component of PICO expression: (P) Population, individuals with Type 2 diabetes or obesity; (I) Intervention, GLP1‐RA; (C) Comparison, placebo, and (O) Outcomes, HF events. The PubMed search was done on October 12, 2024, and included the following terms: ((“diabetes mellitus”[Mesh Terms] OR “type 2 diabetes”) OR (“obesity”[Mesh Terms])) AND (“Glucagon‐Like Peptide 1”[MeSH Terms] OR GLP1RA OR “glucagon‐like peptide 1 receptor agonists” OR “glucagon‐like peptide‐1 receptor agonist”) AND (“placebos”[MeSH Terms] OR placebo) AND (“randomized controlled trial” [Publication Type] OR “randomized controlled trial” OR random*[Title/Abstract]) AND (“heart failure”) NOT ((“meta‐analysis”[Publication Type]) OR (“review”[Publication Type])). We searched MEDLINE (via PubMed) without restricting the search by language, date, or publication status. We identified 68 studies, of which we excluded 62 because of inadequate study design, outcomes, or topic of interest. Then we assessed whether the identified trials included a subgroup without HF at baseline, resulting in six selected RCTs: (1) Liraglutide Effect and Action in Diabetes: Evaluation of Cardiovascular Outcome Results (LEADER; NCT01179048) [17]; (2) Exenatide Study of Cardiovascular Event Lowering Trial (EXSCEL; NCT01144338) [18]; (3) Effect of Albiglutide, When Added to Standard Blood Glucose Lowering Therapies, on Major Cardiovascular Events in Subjects With Type 2 Diabetes Mellitus (HARMONY, NCT02465515) [19]; (4) Researching Cardiovascular Events With a Weekly Incretin in Diabetes (REWIND, NCT01394952) [20]; (5) Semaglutide Effects on Heart Disease and Stroke in Patients With Overweight or Obesity (SELECT; NCT03574597) [21]; (6) Research Study to See How Semaglutide Works Compared with Placebo in People With Type 2 Diabetes and Chronic Kidney Disease (FLOW; NCT03819153) [22].

Two independent reviewers (J.S.N. and J.P.F.) confirmed the eligibility of the included trials. J.S.N. performed data extraction using standardized spreadsheets, with independent verification of the data by A.R.L. and C.V. J.S.N., and J.P.F performed study quality assessment. We assessed the risk of bias in randomized trials with the RoB 2 tool [23]. We also cross‐checked the RCTs selected for the present analysis with other previously published meta‐analyses and the concordance was perfect [15, 24]. We assessed publication bias by visual inspection of funnel plots, with ascertainment for potential asymmetry of published results using Egger's regression test.

For most trials, participants were classified as having or not having HF by study investigators. Unlike the other trials, REWIND included two different definitions of HF: having HF at baseline and having a history of “prior heart failure”. Only outcomes for those without HF at baseline were included. Because of this definition, some participants in the “no baseline HF” group had a history of prior HF (10.2%). To exclude potential bias related to this definition, we performed a sensitivity analysis where we excluded the REWIND trial.

### 2.2 Outcomes

The primary outcome was the time to first HF event (defined as HF hospitalization or urgent visit for HF). The secondary outcome was the time to a composite of an HF event or cardiovascular death.

HF events and cardiovascular death were independently adjudicated in all trials, except in the FLOW trial where only cardiovascular death was adjudicated. In the LEADER, HARMONY Outcomes and EXSCEL trials, only HF hospitalizations were reported; therefore, for these trials, the outcome HF event does not include urgent visits for HF.

### 2.3 Data Analysis

A random‐effect meta‐analysis approach was used with heterogeneity assessed using the Cochran Q test statistic and the “Higgins and Thompson I^2^” [25]. Heterogeneity was considered to be low, moderate, or high if I^2^ was < 25%, 25% to 75%, or > 75%, respectively. Estimates from each study were combined by use of inverse variance‐weighted averages of logarithmic HR in random‐effects analysis.

The hazard ratio (HR) and the respective 95% confidence intervals (95% CI) were extracted from the treatment effect estimates in the subgroups without HF. In the SELECT trial, the HR and 95% CI for HF event was not reported in the subgroup without HF. However, the number of events was available, and we calculated the odds ratio for this outcome and used it for the main analysis. As a sensitivity analysis, we performed the meta‐analysis excluding SELECT for the HF events outcome.

We performed univariate meta‐regression analyses to estimate the relationship between log‐transformed HR for HF events and either body weight loss, HbA1c decrease, SBP variation, DBP variation, heart rate increase, or the log‐transformed HR for MACE. When available, we obtained these variables from the subgroup without HF at baseline in each trial. When subgroup‐level data were not available, we obtained the variation in each predictor variable from the total population (Table S1).

We also compared treatment effect on HF events according to the following trial characteristics: ASCVD status (all patients with ASCVD vs. multiple risk factors or ASCVD), HF incidence rate in the placebo (< 1 event vs. ≥ 1 event per 100 patients‐year), mean baseline HbA1c (< 8% vs. ≥ 8%), and type of GLP‐1 RA (semaglutide vs. other GLP‐1 RAs).

Because of the limited precision of the available HR and 95% CI (i.e., just two decimal places), a CI tolerance of 0.05 was required to run this meta‐analysis. We considered statistically significant for main effects a two‐sided *p*‐value < 0.05, and for interaction tests, a two‐sided *p*‐value < 0.10, because of the lesser statistical power of the latter. We performed the statistical analyses using Stata software version 18 (StataCorp. 2023. Stata Statistical Software: Release 18. College Station, TX: StataCorp LLC), and registered this meta‐analysis on PROSPERO (CRD42024597320).

### 2.4 Data and Resource Availability

All data is publicly available in the relevant primary and secondary papers from relevant trials as listed in the References.

## 3 Results

### 3.1 Study Selection and Patient Population

Reporting Items for Systematic Reviews and Meta‐analyses (PRISMA) flow chart detailing the selection process is presented in Supplementary Figure S1. From 68 initial reports, we identified six placebo‐controlled RCTs reporting HF outcomes with GLP‐1 RA in patients without HF [17, 18, 19, 20, 21, 22]. In total, 52,752 participants without HF at randomization were included in the six RCTs: 25.2% in SELECT, 23.4% in EXSCEL, 17.1% in REWIND, 14.5% in LEADER, 14.3% in HARMONY, and 5.4% in the FLOW trial (Table 1).

**TABLE 1:** Baseline characteristics of patients without HF across trials.

Trial	LEADER	EXSCEL	HARMONY	REWIND	SELECT	FLOW
ClinicalTrials.gov ID	NCT01179048	NCT01144338	NCT02465515	NCT01394952	NCT03574597	NCT03819153
Total N.	9340	14,752	9463	9901	17,604	3533
Participants without HF, *n* (%)	7673 (82.2)	12,362 (83.8)	7540 (79.7)	9009 (91.4)	13,314 (75.6)	2854 (80.8)
Intervention	Liraglutide up to 1.8 mg daily	Exenatide 2 mg weekly	Albiglutide 30–50 mg weekly	Dulaglutide 1.5 mg weekly	Semaglutide 2.4 mg weekly	Semaglutide 1.0 mg weekly
Median duration of treatment	3.5 years	3.2 (2.2–4.4) years	1.6 (1.3–2.0) years	5.4 (5.1–5.9) years	2.8 ± 1.1 years^a^	3.4 years
Female, *n* (%)	2653 (34.6)	4753 (38.4)	2223 (29.5)	4207 (46.7)	3732 (28.0)	822 (28.8)
Age, years	64.4 ± 7.1	62.0 (55.0–68.0)	65.0 (58.0–70.0)	66.2 ± 6.4	61.5 ± 8.9	67.0 (61.0–73.0)
BMI, kg/m^2^	32.2 ± 6.1	31.6 (28.1–35.9)	31.2 (28.0–35.2)	32.2 ± 5.7	33.1 ± 4.9	30.8 (27.0–34.7)
Type 2 diabetes, *n* (%)	7673 (100)	12,362 (100)	7540 (100)	9009 (100)	0 (0%)	2854 (100)
Diabetes duration, years	13.1 ± 8.0	12.0 (7.0–18.0)	13.0 (7.8–19.5)	9.5 (5.5–14.5)	Not applicable	NR
CKD, *n* (%)	1725 (22.5)^b^	2474 (16.8)	1564 (20.7)^b^	NR	NR	2854 (100)
ASCVD, *n* (%)	NR (81.3%^d^ in the total group)	8714 (70.5)	7540 (100)	2898 (32.2)	13,314 (100)	542 (19.2)^e^
AF/flutter (%)	NR	NR	468 (6.2)	91 (1.0)	NR	142 (5.0)
HbA1c, %	8.7 ± 1.5	8.0 (7.3–8.8)	8.3 (7.6–9.4)	7.3 ± 1.1	5.78 ± 0.34	7.5 (6.8–8.5)
eGFR, mL/min/1.73 m^2^	80.9 ± 27.5	77.2 (62.4–92.9)	78 (62–95)	75.5 (61.9–92.0)	83.4 ± 17.0	45.0 (35.0–57.0)
UACR, mg/g Cr	NR	NR	NR	NR	7.3 (4.5–15.0)	567.7 (234.4–1320.0)
Systolic BP, mmHg	136.1 ± 17.6	NR	135 (124–145)	137.3 ± 16.8	131.5 ± 15.5	138.0 (128.0–148.0)
Metformin, *n* (%)	NR	10,587 (85.6)^c^	5659 (75.1)	NR	NR	1496 (52.4)
Insulin, *n* (%)	NR	5613 (44.7)	4420 (58.6)	2130 (23.6)	0 (0%)	1709 (59.9)
SGLT2 inhibitors, *n* (%)	NR	NR	488 (6.5)	2 (0.0)	NR	468 (16.4)
Beta‐blockers, *n* (%)	3970 (51.7)	6408 (51.8)	4738 (62.8)	3816 (42.4)	8782 (66.0)	1324 (46.4)
ACEi/ARB, *n* (%)	ACEi: 3827 (49.9) ARB: 2465 (32.1)	ACEi: 5780 (46.8) ARB: 3906 (31.6)	5877 (77.9)	7325 (81.3)	ACEi: 5805 (43.6) ARB: 3778 (28.4)	2719 (95.3)
MRA, *n* (%)	155 (2.0)	481 (3.9)	414 (5.5)	272 (3.0)	648 (4.9)	144 (5.0)

*Note:* Baseline characteristics of participants included in this analysis. All percentages presented refer to the subpopulation without heart failure at randomization. Values are *n* (%), mean ± SD or median (interquartile range), unless stated otherwise.

Abbreviations: ACEi—Angiotensin‐converting enzyme inhibitors; AF—Atrial fibrillation; ARB—Angiotensin receptor blockers; ASCVD—Atherosclerotic cardiovascular disease; BMI—Body mass index; CABG—Coronary artery bypass graft; CAD—Coronary artery disease; CKD—Chronic kidney disease; EXSCEL – Exenatide Study of Cardiovascular Event Lowering; FLOW—Research Study To See How Semaglutide Works Compared to Placebo in People With Type 2 Diabetes and Chronic Kidney Disease; HARMONY—Effect of Albiglutide, When Added to Standard Blood Glucose Lowering Therapies, on Major Cardiovascular Events in Subjects With Type 2 Diabetes Mellitus; LEADER – Liraglutide Effect and Action in Diabetes: Evaluation of Cardiovascular Outcome Results; MRA—Mineralocorticoid receptor antagonists; NR—Not reported; PAD—Peripheral arterial disease; REWIND—Researching Cardiovascular Events With a Weekly Incretin in Diabetes; SELECT—Semaglutide Effects on Heart Disease and Stroke in Patients With Overweight or Obesity; TIA—Transient ischemic attack.

^a^ Duration of treatment in the SELECT study is reported as mean ± SD.

^b^ CKD prevalence at baseline is not reported, so figures presented represent the prevalence of eGFR < 60 mL/min/1.73 m^2^.

^c^ Treatment with metformin is not reported; values reported refer to treatment with any oral antidiabetic agents.

^d^ Established cardiovascular disease.

^e^ Prior myocardial infarction or stroke.

The proportion of participants without previous HF in each trial ranged from 76% to 91%. All RCTs included patients with Type 2 diabetes, except the SELECT trial in which Type 2 diabetes diagnosis was an exclusion criterion. In the SELECT and HARMONY trials, all participants had a history of ASCVD; whereas in the other trials participants with Type 2 diabetes and risk factors for cardiovascular disease were also included; all participants in the FLOW trial had CKD (Table 1).

Therapy for Type 2 diabetes differed across the trials as shown in Table 1. The use of SGLT2 inhibitors at baseline was only reported in HARMONY (6.5% of the participants) and FLOW (16.4%). An overall high proportion of participants was taking beta‐blockers (42%–66% of participants) and ACEi/ARB (more than two‐thirds in all trials) at randomization.

All RCTs included in this meta‐analysis had a low risk of bias (Figure S2). Visual inspection of funnel plots and Egger's test did not suggest the presence of publication bias (Figure S3).

### 3.2 Treatment With GLP‐1 RA and HF Events

**FIGURE 1:**
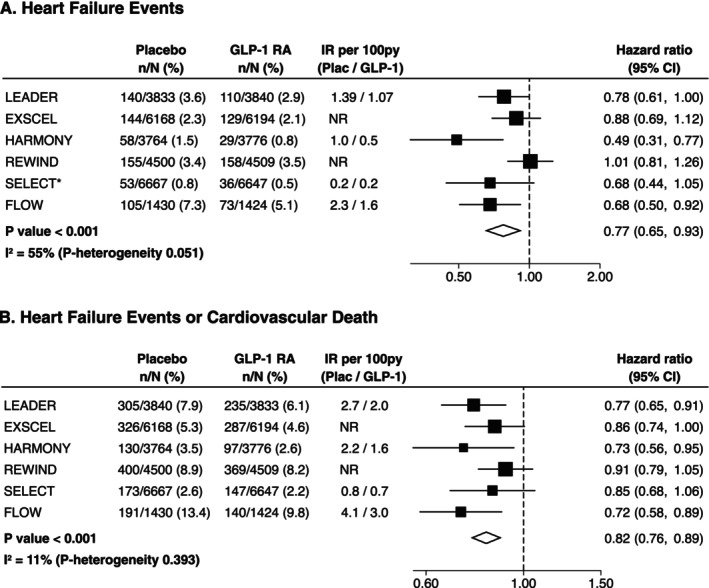
Effects of GLP‐1 RA versus placebo on heart failure events (A) or composite outcome of heart failure events or cardiovascular death (B) in patients without heart failure. Abbreviations: EXSCEL, Exenatide Study of Cardiovascular Event Lowering; FLOW, Research Study To See How Semaglutide Works Compared to Placebo in People With Type 2 Diabetes and Chronic Kidney Disease; HARMONY, Effect of Albiglutide, When Added to Standard Blood Glucose Lowering Therapies, on Major Cardiovascular Events in Subjects With Type 2 Diabetes Mellitus; IR, incidence rate; LEADER, Liraglutide Effect and Action in Diabetes: Evaluation of Cardiovascular Outcome Results; REWIND, Researching Cardiovascular Events With a Weekly Incretin in Diabetes; SELECT, Semaglutide Effects on Heart Disease and Stroke in Patients With Overweight or Obesity.

Treatment with GLP‐1 RA (vs. placebo) decreased the risk of HF events: HR 0.77, 95% confidence interval [CI] 0.65–0.93, *p* < 0.001 (Figure 1A), and the risk of the composite of HF events or cardiovascular death: HR 0.82, 95% CI 0.76–0.89, *p* < 0.001 (Figure 1B).

We observed similar results in the sensitivity analysis excluding the SELECT trial (Figure S4) and the REWIND trial (Figure S5).

### 3.3 Correlation Between Changes in Key Risk Factors and HF Events

**FIGURE 2:**
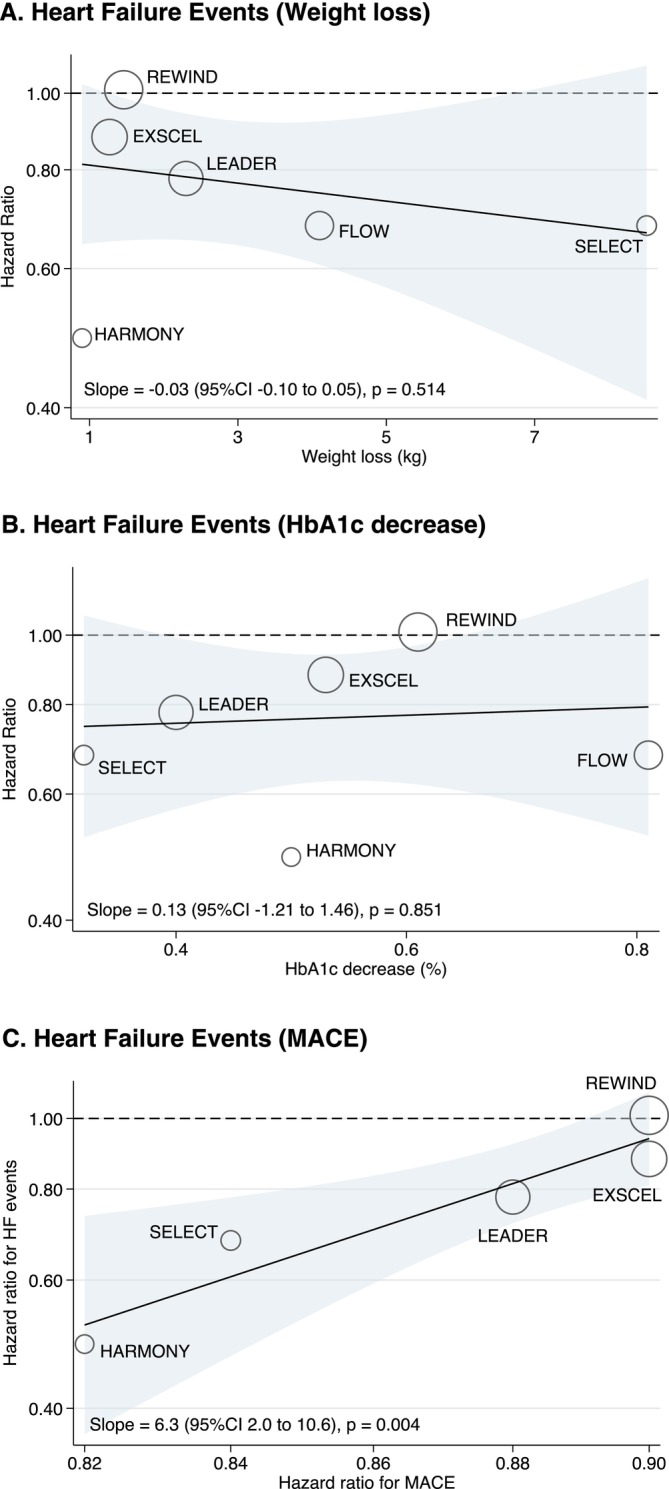
Meta‐regression of the association of log‐transformed hazard ratio for HF events and weight loss (A), HbA1c decrease (B), or log‐transformed hazard ratio for MACE (C). Abbreviations: EXSCEL, Exenatide Study of Cardiovascular Event Lowering; FLOW, Research Study To See How Semaglutide Works Compared to Placebo in People With Type 2 Diabetes and Chronic Kidney Disease; IR, incidence rate; HARMONY, Effect of Albiglutide, When Added to Standard Blood Glucose Lowering Therapies, on Major Cardiovascular Events in Subjects With Type 2 Diabetes Mellitus; HF, heart failure; LEADER, Liraglutide Effect and Action in Diabetes: Evaluation of Cardiovascular Outcome Results; MACE, major atherosclerotic cardiovascular events; REWIND, Researching Cardiovascular Events With a Weekly Incretin in Diabetes; SELECT, Semaglutide Effects on Heart Disease and Stroke in Patients With Overweight or Obesity.

The effect of GLP‐1 RA on HF events was not correlated with weight loss (Figure 2A), HbA1c decrease (Figure 2B) nor changes in blood pressure or heart rate (Figure S6). On the other hand, there was a significant correlation between the HR of GLP‐1 RA (vs. placebo) for HF events and for MACE (Figure 2C).

### 3.4 Effects of GLP‐1 RA on HF Events According to Patients' Characteristics

**FIGURE 3:**
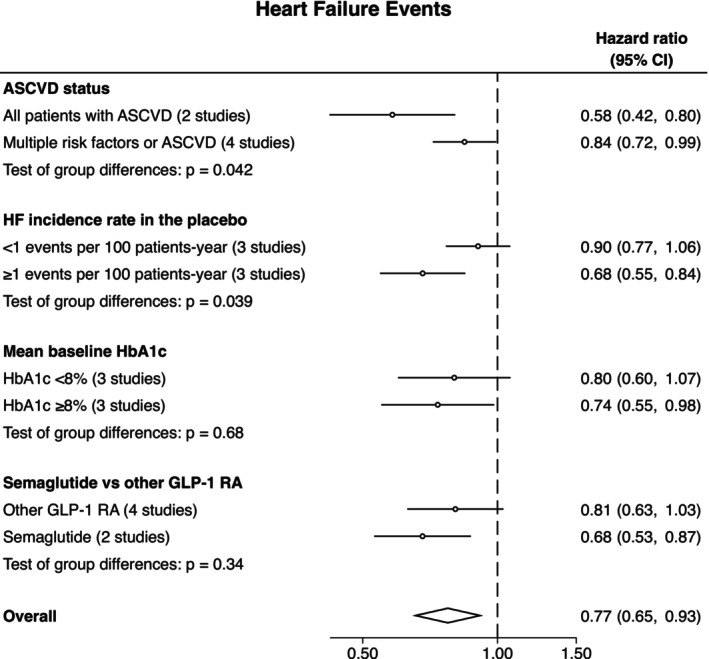
Subgroup analyses by trial characteristics. Abbreviations: ASCVD, atherosclerotic cardiovascular disease; HF, heart failure.

The effects of GLP‐1 RA on HF events were more pronounced in studies that restricted inclusion to participants with previous ASCVD (vs. also including participants with multiple risk factors): HR 0.58 (95% CI 0.42–0.80) vs. HR 0.84 (0.72–0.99), *p*‐interaction = 0.042. Similarly, the effects of GLP‐1 RA on HF events were more pronounced in studies with higher incidence rate of HF events (≥ 1 event vs. < 1 event per 100 patients‐year): HR 0.68 (95% CI 0.55–0.84) vs. 0.90 (95% CI 0.77–1.06), *p*‐interaction = 0.039 (Figure 3).

The effect of GLP‐1 RA on HF events was not modified by the mean baseline HbA1c or the type of GLP‐1 RA (Figure 3).

## 4 Discussion

In this meta‐analysis of RCTs in people with Type 2 diabetes or obesity without known HF, treatment with a GLP‐1 RA reduced the risk of HF events by 23%, suggesting a protective effect of GLP‐1 RA on the risk of new‐onset HF.

These results are concordant with previous studies suggesting a preventive effect of GLP‐1 RAs on new‐onset HF. An observational cohort study set in England and Wales documented that, in 411,206 individuals with Type 2 diabetes and without HF, treatment with GLP‐1 RA reduced the risk of HF events by 18% [26]. In our prior meta‐analysis of four RCTs in people with Type 2 diabetes, treatment with GLP‐1 RAs was associated with a decreased risk of HF events in those without HF but not in those with previous history of HF [15]. The previous meta‐analysis was limited by a smaller total study population (36,625 vs. 52,752 in the current meta‐analysis), the inclusion of only participants with Type 2 diabetes, and the absence of meta‐regression analysis to clarify the mechanism of the protective effect. The current meta‐analysis provides valuable new information by showing that the protective effect on new‐onset HF is also observed in people with obesity without Type 2 diabetes, as well as in populations with a high prevalence of CKD. Furthermore, this meta‐analysis indicates that the prevention of new‐onset HF was independent of weight loss and HbA1c decrease but was associated with the effect on MACE.

A protective effect of GLP‐1 RAs was also observed in the GRADE trial. The GRADE (Glycemia Reduction Approaches in Type 2 Diabetes: A Comparative Effectiveness) trial targeted patients with relatively low cardiovascular risk (i.e., patients with Type 2 diabetes of less than 10 years' duration, only on metformin treatment and with glycated hemoglobin levels of 6.8%–8.5%) [27]. These participants were randomized to one of four interventions: a DPP‐4 inhibitor (sitagliptin), a sulfonylurea (glimepiride), insulin glargine, or a GLP‐1 RA (liraglutide) [27]. Liraglutide reduced the risk of HF hospitalization by 51%, compared with other interventions [27]. Whether this was due to a protective effect of liraglutide, an adverse effect of the comparators, or a combination was unclear. In the present meta‐analysis, only trials comparing GLP‐1 RAs with placebo were included and, in contrast to the GRADE trial, most patients had high cardiovascular risk at baseline.

The pooled analysis of the STEP‐HFpEF and STEP‐HFpEF DM trials [14], and the results from the SUMMIT trial [13] showed a protective effect of GLP‐1 RA on HF events in patients with HFpEF. Conversely, data from RCTs in HFrEF suggest a lack of protective effects of GLP‐1 RA and potential harm in this group [28, 29, 30, 31]. The current meta‐analysis suggests that, in patients without HF, the effects of GLP‐1 RA are more comparable with those observed in populations with HFpEF than to those in HFrEF.

The mechanisms by which GLP‐1 RA would prevent HF are not fully understood. Interestingly, the benefit of GLP‐1 RAs in preventing HF appears to be independent of weight loss, reductions in HbA1c, and changes in blood pressure. Our results indicate that GLP‐1 RA may prevent HF events even in patients who do not experience significant weight loss or notable improvements in HbA1c with GLP‐1 RA. Evidence from genetic studies suggests that GLP‐1 receptor activation could help prevent HF. GLP‐1 receptors are expressed in both atrial and ventricular cardiomyocytes as well as in endothelial cells within the human heart [32]. Daghlas et al. demonstrated that genetic variants associated with increased GLP‐1R activity were linked to a reduced risk of HF and improved left ventricular ejection fraction [33].

The current analysis suggests that the prevention of HF events with GLP‐1 RA is linked to their impact on reducing atherosclerotic events. GLP‐1 RAs appear to have anti‐atherogenic effects, potentially mediated by improvements in endothelial function and stabilization of atherosclerotic plaques [34], and/or through systemic and cardiac inflammation reduction [35]. These mechanisms likely contribute to weight‐independent cardiometabolic benefits. The extent to which these effects impact the prevention of HF remains to be explored.

Our results also suggest that the protective effect may be greater among individuals with a history of ASCVD or those with higher baseline risk of HF events. These patients face a particularly high risk of developing HF, which may represent a window of opportunity for early treatment with GLP‐1 RA. As discussed above, findings from the GRADE trial indicate that the benefits of GLP‐1 RA in preventing HF may also extend to those with lower cardiovascular risk [27]. From a clinical perspective, the results from the GRADE trial and from the current meta‐analysis suggest that for patients requiring treatment intensification, using a GLP‐1 RA rather than other interventions (excluding SGLT2 inhibitors), may reduce the risk of developing HF.

In most trials included in this analysis, the use of SGLT2 inhibitors was limited, as they were conducted before these drugs became widely available. Previous meta‐analyses have shown a reduction in the risk of HF events with SGLT2 inhibitors across the spectrum of cardiometabolic disease, including those without known HF [36]. It remains uncertain whether the reduction in HF events with GLP‐1 RAs would still be observed in a population with greater use of SGLT2 inhibitors. In a meta‐analysis of Harmony Outcomes and AMPLITUDE‐O (Effect of Efpeglenatide on Cardiovascular Outcomes), a comparison of cardiovascular outcomes with GLP‐1 RAs in patients with and without SGLT2 inhibitor treatment showed a reduction in HF events both in those without SGLT2 inhibitors (HR 0.72, 95% CI 0.55–0.92) and in those treated with SGLT2 inhibitors (HR 0.34, 95% CI 0.12–0.96) [37].

The current meta‐analysis did not include GLP‐1‐based multi‐agonists. Whether these agents will provide a similar, or even greater, benefit in preventing HF events remains uncertain. In the SUMMIT (A Study of Tirzepatide in Participants With Heart Failure With Preserved Ejection Fraction and Obesity: The SUMMIT Trial) [38], in patients with obesity and established HFpEF, tirzepatide (vs. placebo) led to a lower risk of a composite of death from cardiovascular causes or worsening HF and improved health status. The upcoming SURPASS‐CVOT (A Study of Tirzepatide Compared With Dulaglutide on Major Cardiovascular Events in Participants With Type 2 Diabetes) [39], which compares cardiovascular outcomes in high‐risk patients randomized to tirzepatide (a dual GLP‐1/GIP receptor agonist) or dulaglutide (a GLP‐1 RA) in patients with Type 2 diabetes, may provide valuable insights into the effects of GLP‐1‐based multi‐agonists versus GLP‐1 RA on HF prevention. Although the trial's primary outcome is MACE rather than HF, the secondary endpoint of HF hospitalization may still yield clinically relevant information.

This study has some limitations. First, most trials were not originally designed to evaluate HF outcomes and used different definitions for HF events. Second, the definition of previous diagnosis of HF varied between trials and some patients may have had undiagnosed HF at baseline. In the REWIND trial, the group without HF at baseline included patients with a prior history of HF [20]. This may explain why the REWIND trial had a neutral HR for HF events with GLP‐1 RA treatment. Nevertheless, the preventive effect on new‐onset HF was observed even with the inclusion of REWIND in the meta‐analysis, which could potentially underestimate the protective effect. Third, for the SELECT trial, the HR for our primary outcome was not reported, which led to the calculation of the odds ratio (OR). While the OR likely reflects a similar trend to the HR, it may be less precise in capturing the full impact of the drugs. Nevertheless, the sensitivity analysis excluding the SELECT trial also demonstrated a protective effect of GLP‐1 RA in HF events.

## 5 Conclusions

GLP‐1 RA reduce the risk of new‐onset HF in patients with Type 2 diabetes or obesity without prior history of HF. From a clinical perspective, these results suggest that GLP‐1 RA, in addition to their well‐established benefits on weight loss, HbA1c decrease and protection from atherosclerotic events, may be used for the prevention of new‐onset HF in high‐risk patients.

## Conflicts of Interest

J.S.N. reports having received consulting or speaker fees from AstraZeneca, Bayer, BIAL, Boehringer Ingelheim, Eli Lilly, Novo Nordisk, Menarini and Merck. C.B.L. has received research support from the Danish Diabetes Academy (Novo Nordisk Foundation) and from Dexcom. F.V.N. has received consulting or speaker fees from AstraZeneca, Bayer, Daiichi Sankyo and Ultragenyx. A.L.M. has received research support from Boehringer‐Ingelheim, AstraZeneca and Novartis. J.J.H. has in the recent three years been an advisory/consultant for, been on the advisory board of, and/or received research support from Eli Lilly, Novo Nordisk, Zealand Pharma, MSD Denmark, Structure Therapeutics, Scohia., HealthCap, Morgan Stanley, MEDACorp Inc., Arix Bioscience, Alphasights, Alcimed, Google Ventures Management, Guidepoint, Tema, Jefferies International Limited, Amgen, Sofinnova Partners, Thinks Insight & Strategy, AstraZeneca, Septerna; is co‐founder of Antag Therapeutics and Villus (Bainan Biotech). J.P.F. has received research support from Boehringer‐Ingelheim, AstraZeneca, Novartis, Bayer, Salamandra, Bial, Abbott.

## Supporting information


**Table S1:** OBR_70043‐sup‐0001‐Online‐OnlySupplementalMaterial.pdf. Variation of weight, HbA1c, blood pressure and heart rate; and hazard ratio for Major Atherosclerotic Cardiovascular Events in the included trials (GLP‐1 RA vs placebo).
**Figure S1:** PRISMA flow diagram of included trials.
**Figure S2:** Assessment of bias among trials included in meta‐analysis using the Cochrane risk of bias tool RoB 2 for randomized trials. Risk of bias plot created with robvis. McGuinness, LA, Higgins, JPT. Risk‐of‐bias VISualization (robvis): An R package and Shiny web app for visualizing risk‐of‐bias assessments. Res Syn Meth. 2020; 1–7. https://doi.org/10.1002/jrsm.1411.
**Figure S3:** Funnel plot for the worsening HF events outcome. Egger test *p*‐value = 0.65.
**Figure S4:** Effects of GLP‐1 RA versus placebo on heart failure events excluding the SELECT trial, which did not present hazard ratio for heart failure events. Abbreviations: EXSCEL, Exenatide Study of Cardiovascular Event Lowering; FLOW, Research Study To See How Semaglutide Works Compared to Placebo in People With Type 2 Diabetes and Chronic Kidney Disease; IR, incidence rate; HARMONY, Effect of Albiglutide, When Added to Standard Blood Glucose Lowering Therapies, on Major Cardiovascular Events in Subjects With Type 2 Diabetes Mellitus; LEADER, Liraglutide Effect and Action in Diabetes: Evaluation of Cardiovascular Outcome Results; REWIND, Researching Cardiovascular Events With a Weekly Incretin in Diabetes; SELECT, Semaglutide Effects on Heart Disease and Stroke in Patients With Overweight or Obesity.
**Figure S5:** Effects of GLP‐1 RA versus placebo on heart failure events excluding the REWIND trial, which included patients with previous history of heart failure in the group classified as not having heart failure at baseline. Abbreviations: EXSCEL, Exenatide Study of Cardiovascular Event Lowering; FLOW, Research Study To See How Semaglutide Works Compared to Placebo in People With Type 2 Diabetes and Chronic Kidney Disease; IR, incidence rate; HARMONY, Effect of Albiglutide, When Added to Standard Blood Glucose Lowering Therapies, on Major Cardiovascular Events in Subjects With Type 2 Diabetes Mellitus; LEADER, Liraglutide Effect and Action in Diabetes: Evaluation of Cardiovascular Outcome Results; REWIND, Researching Cardiovascular Events With a Weekly Incretin in Diabetes; SELECT, Semaglutide Effects on Heart Disease and Stroke in Patients With Overweight or Obesity.
**Figure S6:** Meta‐regression of the association of logtransformed hazard ratio for HF events and variation of systolic blood pressure (A), diastolic blood pressure (B), or heart rate (C). Abbreviations: DBP, diastolic blood pressure; HR, heart rate; SBP, systolic blood pressure.

## Data Availability

The data that support the findings of this study are available from the corresponding author upon reasonable request.
