# Supplementary material for: GLP‐1 Receptor Agonists for the Prevention of New‐Onset Heart Failure: A Systematic Review and Meta‐Analysis of Placebo‐Controlled Randomized Clinical Trials
Source: Obes Rev. 2025 Nov 25;27(4):e70043. doi: 10.1111/obr.70043 (PMC13008604; doi:10.1111/obr.70043)
Supplement: Supplementary file 1 — Table S1: OBR_70043‐sup‐0001‐Online‐OnlySupplementalMaterial.pdf. Variation of weight, HbA1c, blood pressure and heart rate; and hazard ratio for Major Atherosclerotic Cardiovascular Events in the included trials (GLP‐1 RA vs placebo). Figure S1: PRISMA flow diagram of included trials. Figure S2: Assessment of bias among trials included in meta‐analysis using the Cochrane risk of bias tool RoB 2 for randomized trials. Risk of bias plot created with robvis. McGuinness, LA, Higgins, JPT. Risk‐of‐bias VISualization (robvis): An R package and Shiny web app for visualizing risk‐of‐bias assessments. Res Syn Meth. 2020; 1–7. https://doi.org/10.1002/jrsm.1411. Figure S3: Funnel plot for the worsening HF events outcome. Egger test p‐value = 0.65. Figure S4: Effects of GLP‐1 RA versus placebo on heart failure events excluding the SELECT trial, which did not present hazard ratio for heart failure events. Abbreviations: EXSCEL, Exenatide Study of Cardiovascular Event Lowering; FLOW, Research Study To See How Semaglutide Works Compared to Placebo in People With Type 2 Diabetes and Chronic Kidney Disease; IR, incidence rate; HARMONY, Effect of Albiglutide, When Added to Standard Blood Glucose Lowering Therapies, on Major Cardiovascular Events in Subjects With Type 2 Diabetes Mellitus; LEADER, Liraglutide Effect and Action in Diabetes: Evaluation of Cardiovascular Outcome Results; REWIND, Researching Cardiovascular Events With a Weekly Incretin in Diabetes; SELECT, Semaglutide Effects on Heart Disease and Stroke in Patients With Overweight or Obesity. Figure S5: Effects of GLP‐1 RA versus placebo on heart failure events excluding the REWIND trial, which included patients with previous history of heart failure in the group classified as not having heart failure at baseline. Abbreviations: EXSCEL, Exenatide Study of Cardiovascular Event Lowering; FLOW, Research Study To See How Semaglutide Works Compared to Placebo in People With Type 2 Diabetes and Chronic Kidney Disease; IR, incid [file OBR-27-e70043-s001.pdf]

Supplementary Material

GLP-1 receptor agonists for the prevention of new-onset heart failure: a meta-analysis of placebo-controlled randomized clinical trials

Supplementary Table 1. Variation of weight, HbA1c, blood pressure and heart rate; and hazard ratio for Major Atherosclerotic Cardiovascular Events in the included trials (GLP-1 RA vs placebo)

	LEADER		EXSCEL		HARMONY		REWIND		SELECT		FLOW	
	No HF subgroup	Total population	No HF subgroup	Total population	No HF subgroup	Total population	No HF subgroup	Total population	No HF subgroup	Total population	No HF subgroup	Total population
Weight loss, kg	NR	**2.3**	NR	**1.27**	**0.9**	0.83	NR	**1.46**	NR	**8.51**	NR	**4.1**
HbA1c decrease, %	NR	**0.40**	NR	**0.53**	**0.5**	0.52	NR	**0.61**	NR	**0.32**	NR	**0.81**
SBP variation, mmHg	NR	**-1.2**	NR	**-1.57**	**-0.7**	NR	NR	**-1.70**	NR	**-3.31**	NR	**-2.24**
DBP variation, mmHg	NR	**0.6**	NR	**0.25**	NR	NR	NR	**0.12**	NR	**-0.55**	NR	**0.78**
HR variation, bpm	**3.1**	3.0	NR	**2.51**	**1.5**	1.3	NR	**1.87**	NR	**3.1**	NR	NR
MACE, hazard ratio	**0.88**	0.87	**0.90**	0.91	**0.82**	0.78	**0.90**	0.88	**0.84**	0.80	NR	NR

Legend: DBP, diastolic blood pressure; HR, heart rate; MACE, major atherosclerotic cardiovascular events; NR, not reported; SBP, systolic blood pressure. Values highlighted in bold were used for meta-regression (when subgroup-level data was not available, the variation in each predictor variable was obtained from the total population)

Supplementary Figure 1. PRISMA flow diagram of included trials

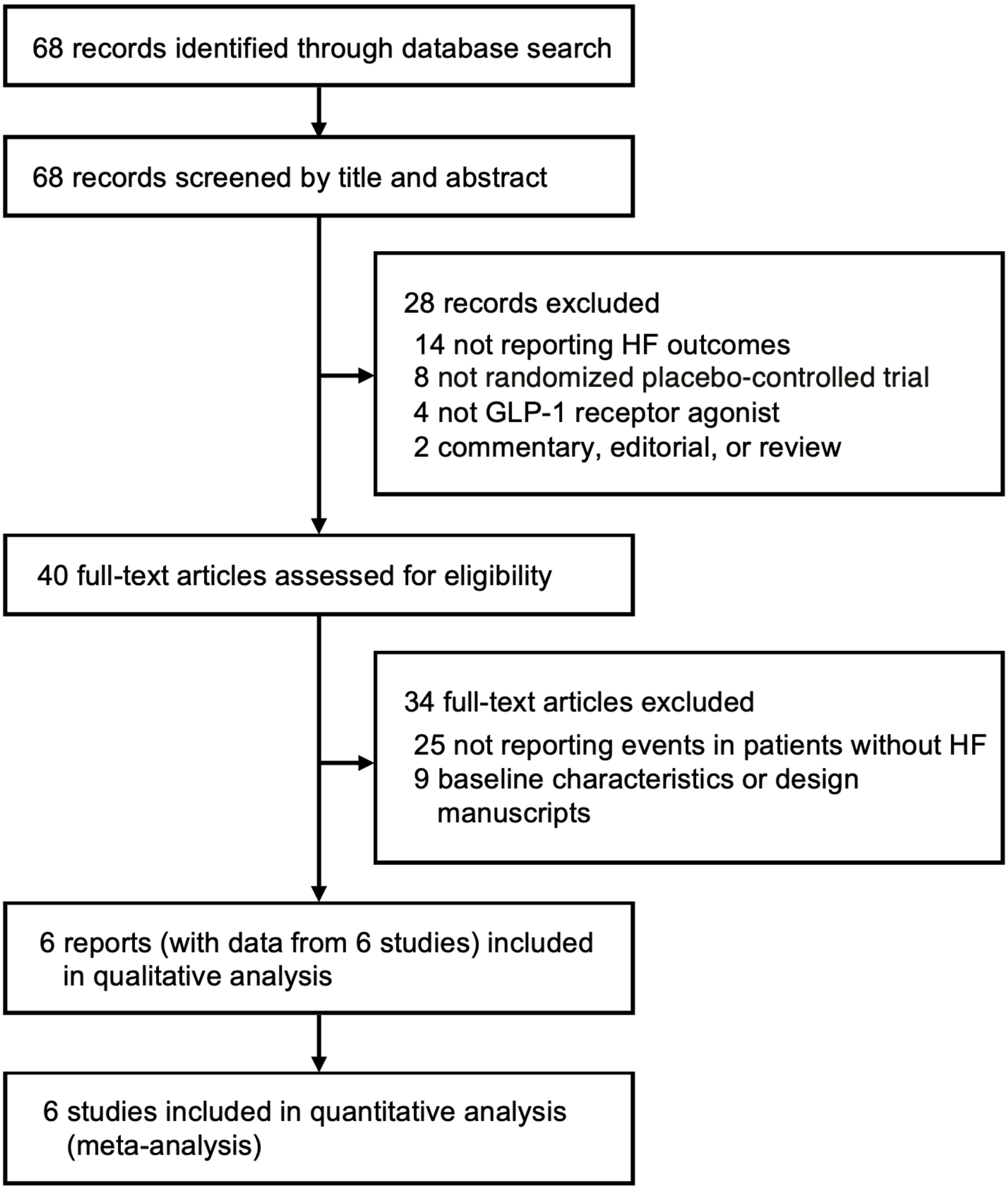

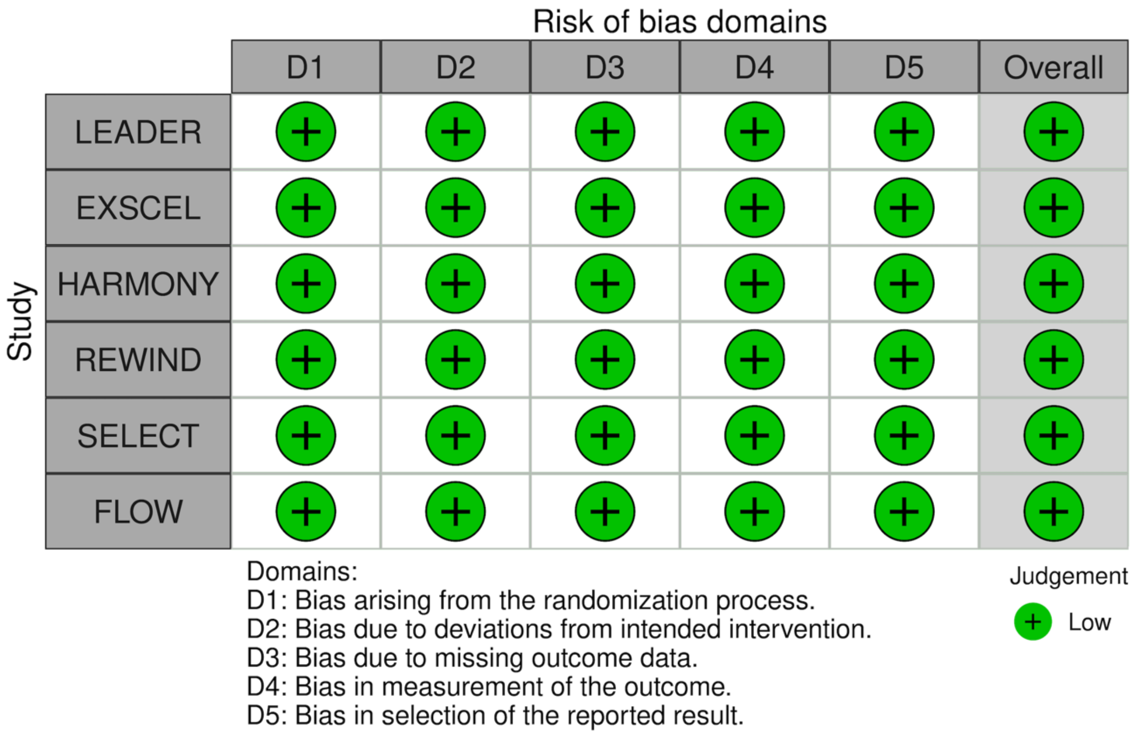

Supplementary Figure 2. Assessment of bias among trials included in meta-analysis using the Cochrane risk of bias tool RoB 2 for randomized trials. Risk of bias plot created with robvis. McGuinness, LA, Higgins, JPT. Risk-of-bias VISualization (robvis): An R package and Shiny web app for visualizing risk-of-bias assessments. Res Syn Meth. 2020; 1- 7. https://doi.org/10.1002/jrsm.1411

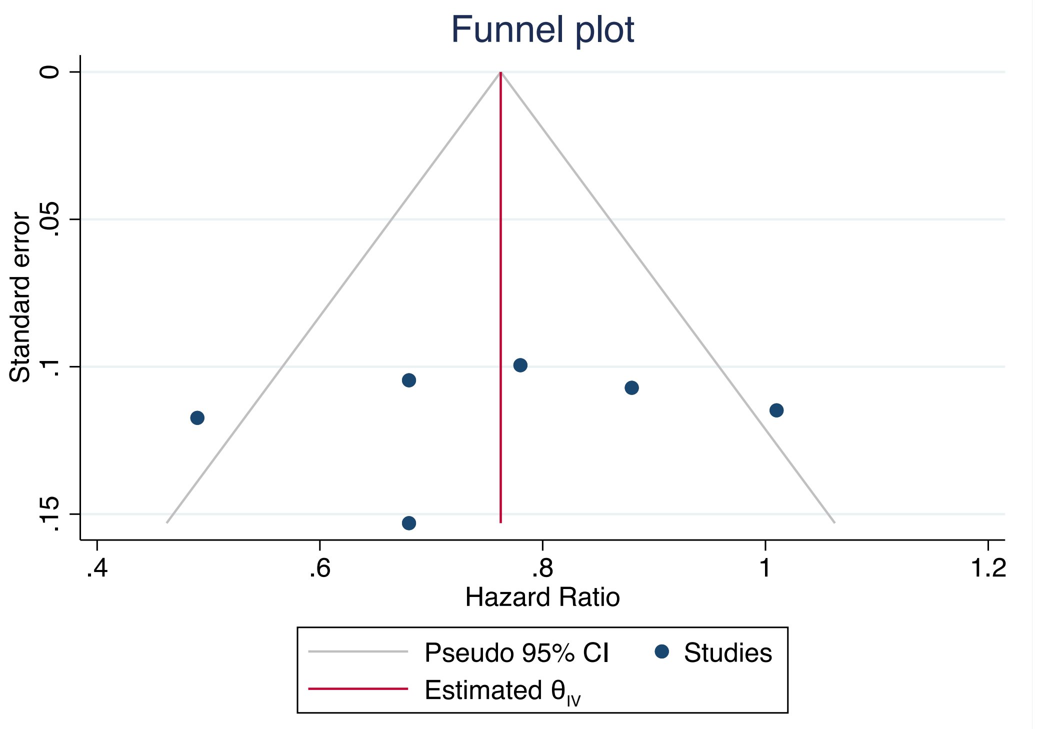

Supplementary Figure 3. Funnel plot for the worsening HF events outcome.

Egger test P-value=0.65.

Heart Failure Events (excluding SELECT)

	Placebo n/N (%)	GLP-1 RA n/N (%)	IR per 100py (P. / GLP-1 RA)	Hazard ratio (95% CI)
LEADER	110/3840 (2.9)	140/3833 (3.6)	1.39 / 1.07	0.78 (0.61, 1.00)
EXSCEL	144/6168 (2.3)	129/6194 (2.1)	NR	0.88 (0.69, 1.12)
HARMONY	58/3764 (1.5)	29/3776 (0.8)	1.0 / 0.5	0.49 (0.31, 0.77)
REWIND	155/4500 (3.4)	158/4509 (3.5)	NR	1.01 (0.81, 1.26)
FLOW	105/1430 (7.3)	73/1424 (5.1)	2.3 / 1.6	0.68 (0.50, 0.92)
**P value < 0.001**				0.78 (0.64, 0.96)
**I^2 = 63% (P-heterogeneity 0.035)**				

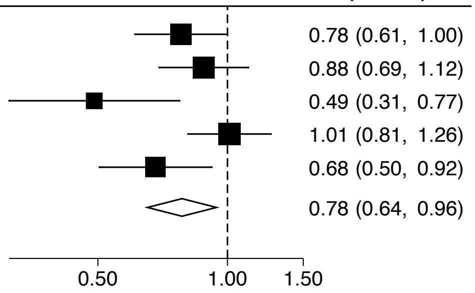

Supplementary Figure 4. Effects of GLP-1 RA versus placebo on heart failure events excluding the SELECT trial, which did not present hazard ratio for heart failure events. Abbreviations: EXSCEL, Exenatide Study of Cardiovascular Event Lowering; LEADER, Liraglutide Effect and Action in Diabetes: Evaluation of Cardiovascular Outcome Results; FLOW, Research Study To See How Semaglutide Works Compared to Placebo in People With Type 2 Diabetes and Chronic Kidney Disease; IR, incidence rate; HARMONY, Effect of Albiglutide, When Added to Standard Blood Glucose Lowering Therapies, on Major Cardiovascular Events in Subjects With Type 2 Diabetes Mellitus; REWIND, Researching Cardiovascular Events With a Weekly Incretin in Diabetes; SELECT, Semaglutide Effects on Heart Disease and Stroke in Patients With Overweight or Obesity

Heart Failure Events (excluding REWIND)

	Placebo n/N (%)	GLP-1 RA n/N (%)	IR per 100py (P. / GLP-1 RA)	Hazard ratio (95% CI)
LEADER	110/3840 (2.9)	140/3833 (3.6)	1.39 / 1.07	0.78 (0.61, 1.00)
EXSCEL	144/6168 (2.3)	129/6194 (2.1)	NR	0.88 (0.69, 1.12)
HARMONY	58/3764 (1.5)	29/3776 (0.8)	1.0 / 0.5	0.49 (0.31, 0.77)
SELECT*	53/6667 (0.8)	36/6647 (0.5)	0.2 / 0.2	0.68 (0.44, 1.05)
FLOW	105/1430 (7.3)	73/1424 (5.1)	2.3 / 1.6	0.68 (0.50, 0.92)
**P value < 0.001**				0.73 (0.62, 0.86)
**I^2 = 27% (P-heterogeneity 0.214)**				

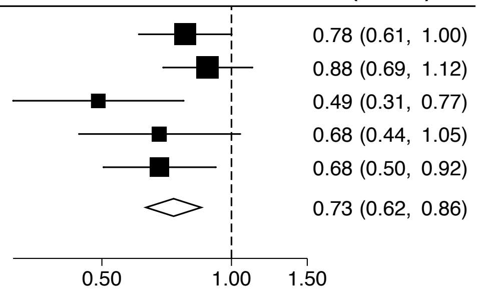

Supplementary Figure 5. Effects of GLP-1 RA versus placebo on heart failure events excluding the REWIND trial, which included patients with previous history of heart failure in the group classified as not having heart failure at baseline. Abbreviations: EXSCEL, Exenatide Study of Cardiovascular Event Lowering; LEADER, Liraglutide Effect and Action in Diabetes: Evaluation of Cardiovascular Outcome Results; FLOW, Research Study To See How Semaglutide Works Compared to Placebo in People With Type 2 Diabetes and Chronic Kidney Disease; IR, incidence rate; HARMONY, Effect of Albiglutide, When Added to Standard Blood Glucose Lowering Therapies, on Major Cardiovascular Events in Subjects With Type 2 Diabetes Mellitus; REWIND, Researching Cardiovascular Events With a Weekly Incretin in Diabetes; SELECT, Semaglutide Effects on Heart Disease and Stroke in Patients With Overweight or Obesity.

A. Heart Failure Events (Systolic Blood Pressure)

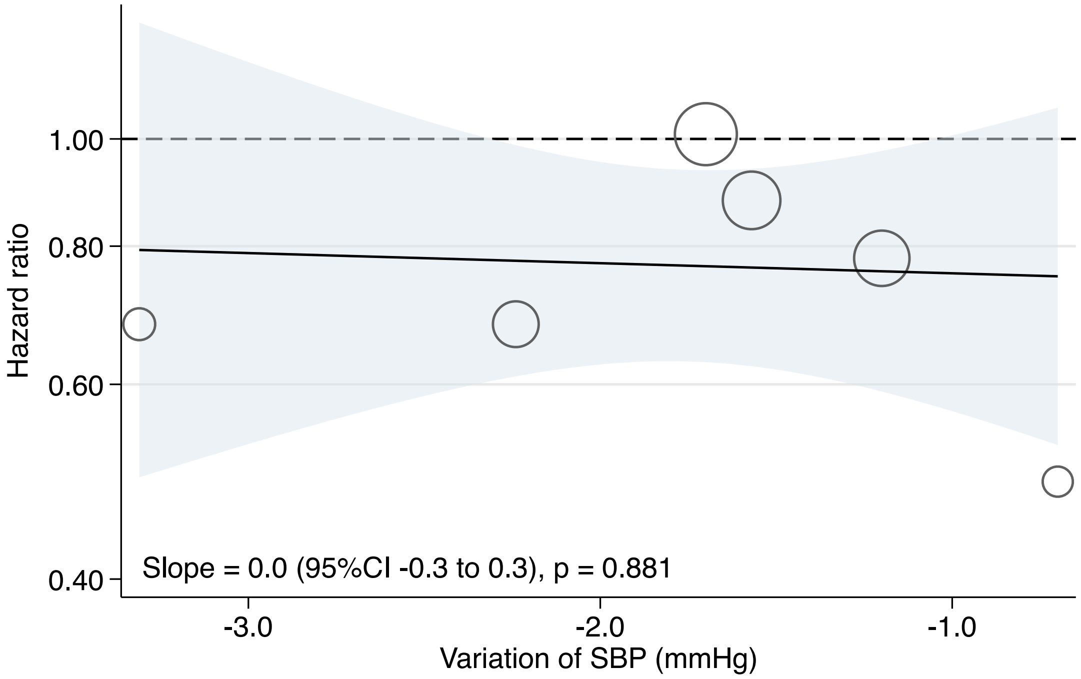

B. Heart Failure Events (Diastolic Blood Pressure)

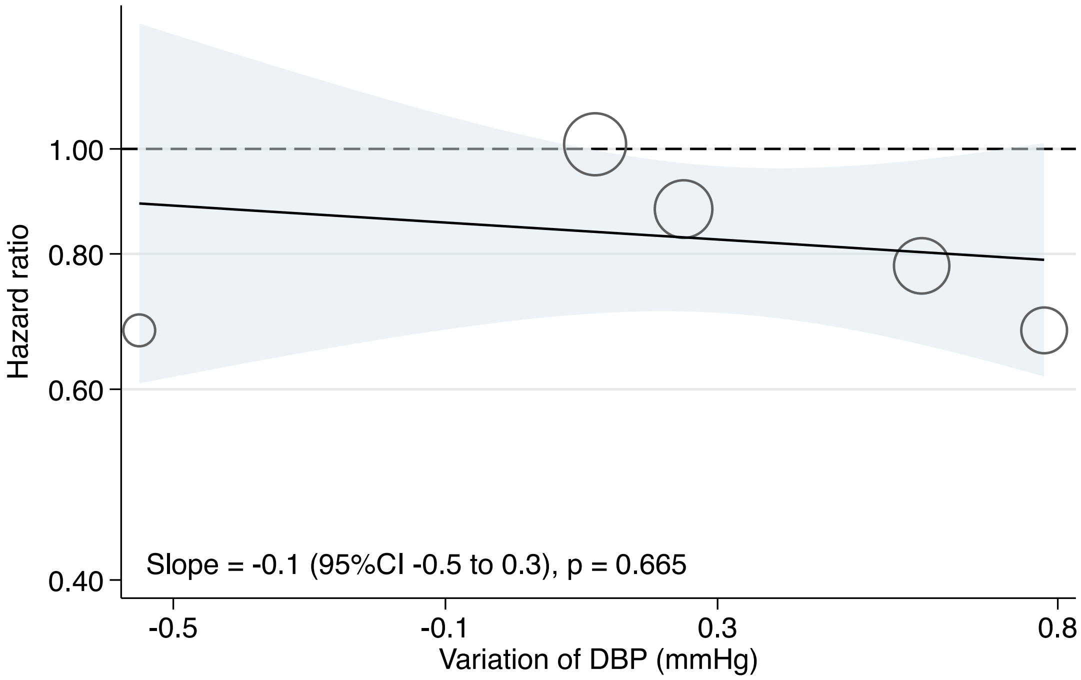

C. Heart Failure Events (Heart Rate)

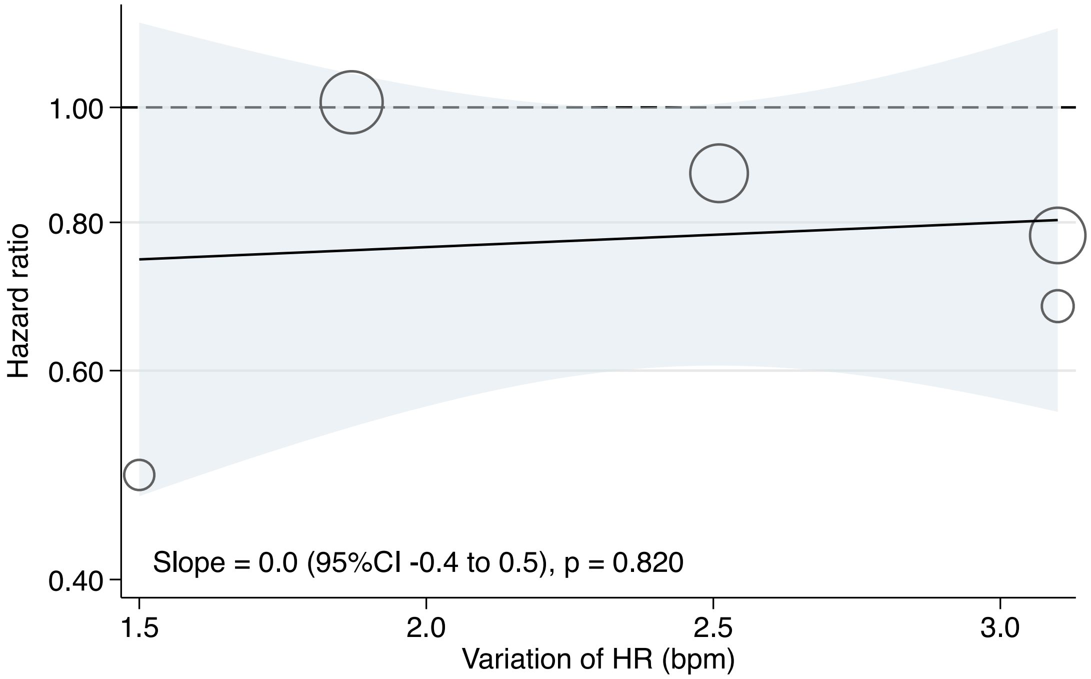

Supplementary Figure 6. Meta-regression of the association of log-transformed hazard ratio for HF events and variation of systolic blood pressure (A), diastolic blood pressure (B), or heart rate (C). Abbreviations: SBP, systolic blood pressure DBP, diastolic blood pressure; HR, heart rate.
